# Antimicrobial and Cell-Penetrating Peptides: Understanding Penetration for the Design of Novel Conjugate Antibiotics

**DOI:** 10.3390/antibiotics11111636

**Published:** 2022-11-16

**Authors:** Andreas Hadjicharalambous, Nikolaos Bournakas, Hector Newman, Michael J. Skynner, Paul Beswick

**Affiliations:** 1 Department of Biochemistry, University of Cambridge, Cambridge CB2 1QN, UK; 2 BicycleTx Limited, Portway Building, Granta Park, Cambridge CB21 6GS, UK; 3 School of Life Sciences, University of Warwick, Coventry CV4 7AL, UK

**Keywords:** antimicrobial peptides, cell-penetrating peptides, membrane active peptides, cell membrane penetration, antimicrobial resistance, peptide characteristics, membrane characteristics, novel antibiotics, peptide-antibiotic conjugates

## Abstract

Antimicrobial peptides (AMPs) are short oligopeptides that can penetrate the bacterial inner and outer membranes. Together with cell-penetrating peptides (CPPs), they are called membrane active peptides; peptides which can translocate across biological membranes. Over the last fifty years, attempts have been made to understand the molecular features that drive the interactions of membranes with membrane active peptides. This review examines the features of a membrane these peptides exploit for translocation, as well as the physicochemical characteristics of membrane active peptides which are important for translocation. Moreover, it presents examples of how these features have been used in recent years to create conjugates consisting of a membrane active peptide, called a “vector”, attached to either a current or novel antibiotic, called a “cargo” or “payload”. In addition, the review discusses what properties may contribute to an ideal peptide vector able to deliver cargoes across the bacterial outer membrane as the rising issue of antimicrobial resistance demands new strategies to be employed to combat this global public health threat.

## 1. Introduction

Membrane active peptides are short natural or synthetic oligopeptides which interact, disrupt and ultimately translocate through biological cell membranes. There are two main classes of membrane active peptides: antimicrobial peptides (AMPs) and cell penetrating peptides (CPPs).

As the name suggests, AMPs contribute to the cell killing of bacteria, viruses and fungi. They are part of the host’s immune defences against pathogens. The first AMP, gramicidin, was discovered from soil microorganisms which were able to lyse gram-positive bacteria [1]. Since then, AMPs have been discovered in a wide range of bacteria, [2] animals [3,4] and plants. [3,5] The majority of AMPs are cationic, made up of short stretches of positively charged amino acids while also having an amphipathic character [6,7]. Although many AMPs have intracellular targets, their initial interaction with the anionic phospholipids of bacterial membranes is an important early step in their direct killing mechanism. Additionally, AMPs can exert their antimicrobial activity indirectly via a secondary immunomodulatory mode of action [6,8].

Cell penetrating peptides (CPPs) are usually between 6 and 30 amino acids in length and are able to penetrate a wide range of biological membranes. The first CPP discovered was the HIV transcription factor *Tat* which was shown to be internalised by human cells, even translocating into the nucleus [9,10]. It was later shown that translocation is possible due to the arginine rich, positively charged region found on the N-terminus of Tat, exclusion of which abolishes uptake [11,12]. Shortly after, the third helix of the *Drosophila* Antennapedia homeoprotein (also called penetratin) was shown to be internalised by neuronal cells, [13] and was shown to be able to carry “cargo” into cells [14,15]. Since then, CPPs have been used as tools to deliver various conjugated cargoes (including fluorophores [12,16,17], DNA [18], nucleic acid derivatives [19,20,21], quantum dots [22,23], proteins [23] and antibiotics [24]) into bacterial, [19,21,23,25] fungal [26] and mammalian [27,28] cells.

AMPs and CPPs share several physicochemical characteristics [29]. The majority of membrane active peptides have an overall positive charge derived from their multiple positively charged residues, such as arginine or lysine, and are amphipathic due to the presence of hydrophobic side chains. Most membrane active peptides have a disordered secondary structure in solution but can assume a more rigid conformation upon membrane interaction [7,29,30,31,32]. Many CPPs have shown antibacterial activity and the majority of AMPs studied are cell penetrating, [6,33] with some showing selectivity for only prokaryotic membranes and some no selectivity between eukaryotic and prokaryotic membranes [30,34,35,36,37,38]. As mentioned above, some AMPs have additional immunomodulatory activity which is a feature of their indirect antimicrobial action, [6,8] and thought to be conducted by direct or indirect immune cell membrane receptor interactions [8,39]. This review focuses on the ability of AMPs to directly penetrate cell membranes. Typically, their pathway across the membrane does not depend on specific interactions with proteins such as receptors and channels, but rather interactions with the membrane. The majority of AMPs have equivalent cell penetration (inner and outer membrane) and direct cell killing in both their all -*D*- and all-*L*- enantiomers, indicating that the interactions needed for penetration are not dependent on the specific (chiral) sequence motif but rather their physicochemical characteristics [40,41,42,43,44,45,46,47].

Research around membrane active peptides has intensified [6,7] not only as an invaluable basic research tool in delivering different compounds intracellularly but also as translational research, as they could be used as “vectors” for anti-microbial [7,21,36,37] or anti-cancer [7,48] drugs or other cargoes. To date, thousands of membrane active peptides have been investigated from all kingdoms of life [7,9,13,35,49,50,51,52]. Several studies have used computational approaches to design in silico membrane active peptides and have been extensively reviewed [29,53,54,55]. The chemical features differentiating membrane active peptides from general oligopeptides are not rigidly defined, due to a lack of complete understanding of how they interact with membranes and likely due to the lack of a single general mechanism driving cell penetration. Nevertheless, there are general features which are important in the ability of the majority of membrane active peptides to penetrate membranes.

This review aims to describe the major aspects, regarding target membranes and peptides, that should be taken into consideration when choosing, modifying or designing a membrane penetrating peptide for transporting cargoes into a bacterial cell. The literature on membrane active peptides is large and their mode of action varied. This review aims to avoid specific descriptions of different classes of AMPs and CPPs as this has been extensively reviewed elsewhere [2,3,4,5,7]. Instead, both the activity-determining features of effective membrane active peptides and ways to incorporate these into new antimicrobial therapeutics is presented. The review discusses briefly the most utilised methods for the study of membrane active peptides. It then focuses on the membrane components that allow peptide penetration and the general physicochemical characteristics of a penetrating peptide that need to be considered when designing new permeators. Finally, the current strategies employed which utilise the features described to incorporate membrane active peptides as conjugates into novel classes of antibiotics to address the rising issue of antimicrobial resistance are presented and discussed.

## 2. Mechanisms of Membrane Translocation by Membrane Active Peptides

The mechanism of membrane translocation is highly dependent on the type of membrane and membrane active peptide. Active uptake mechanisms such as endocytosis and pinocytosis have been proposed as the translocation route of some CPPs and are discussed elsewhere [23,24,56,57,58,59,60,61,62]. Proposed mechanisms of direct (non-active) CPP and AMP translocation include the inverted micelle, the carpet-like and two pore formation models (toroidal or barrel-stave) [6,7,16,63,64,65,66,67].

The inverted micelle model was proposed by De Rossi et al. while studying the penetration mechanism of penetratin [16]. In this model, the peptide interacts with the outer leaflet of the membrane, causing positive curvature, which eventually leads to the formation of a micelle whose lumen consists of phosphate heads interacting with the positively charged side chains of the CPP. This allows an energetically favourable transition of a charged CPP through the membrane. The micelle is eventually disrupted, allowing the release of the peptide [68].

**Figure 1:**
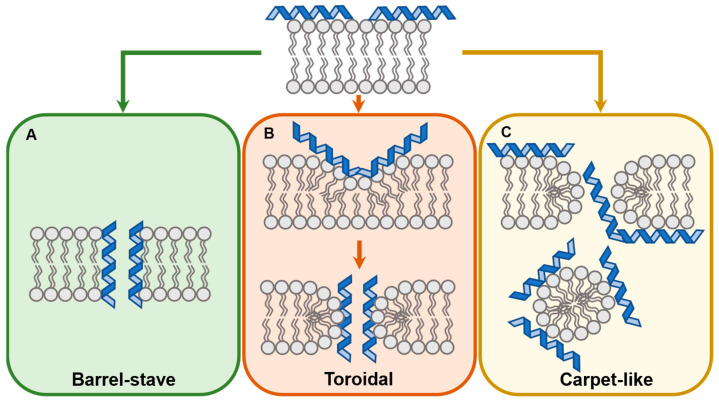
Proposed mechanisms of membrane penetration. The initial interaction of a membrane active peptide occurs with the phosphate heads of the outer leaflet of the membrane and is electrostatic. In the barrel-stave model (**A**) the peptide lines perpendicularly along the membrane forming a pore and interacting with the phosphate heads and the fatty acyl chains. In the toroidal pore model (**B**), the peptide induces membrane deformations, causing the temporary fusion of the inner and outer leaflets. The peptide interacts with the phosphate heads. In the carpet-like model (**C**), membrane active peptides cause the disruption of the membrane in a concentration dependent manner. When this critical concentration is reached, membrane-peptide micelles form leading to disruption of the bilayer.

Proposed by Pony et al., initially studying dermaseptin, the carpet-like model suggests that penetration is achieved by the bulk disruption of the membrane once a critical peptide concentration interacts with the outer leaflet of the membrane (Figure 1C) [66]. The concentration-dependent accumulation of the peptide causes changes in the fluidity and integrity of the membrane, leading to its breakdown and formation of peptide-membrane micelles [6,63,64,66].

The two pore-forming models suggest that CPPs insert into the membrane to form pores and eventual entry of CPPs from the pore lumen. The “barrel-stave” model (Figure 1A) postulates that CPPs disrupt the phosphate heads of the outer leaflets and insert themselves perpendicularly to the membrane. The hydrophobic side chains of the CPP interact with the lipid tails while the charged side chains face the lumen of the pore or interact with the phosphate heads. In the “toroidal-pore” model (Figure 1B) the CPPs are initially in parallel with the membrane but eventually reorient themselves perpendicularly. This causes the thinning and the curving of the outer leaflet which eventually leads to the transient fusion of the inner and outer leaflet at the point where a toroidal, “donut-shaped” pore forms. This transition state is unstable, leading to its disintegration and displacement of some of the CPP molecules onto the inner side of the membrane [6,7,64].

## 3. In Vitro Assays for Characterisation of Membrane Active Peptides

Tools to characterise the ability of peptides to penetrate membranes and deliver their cargoes into the cell are needed to understand how they penetrate cells. Various biochemical and biophysical techniques have been described in the literature to characterise the properties of membrane active peptides. An ideal assay for the activity of a membrane active peptide is sensitive, low cost and accurately reports the accumulation of the cargo inside the cell, with those cells being under near-physiological conditions. No assay with these features has been reported, requiring a series of orthogonal assays to fully understand the specific peptide’s behaviour. This is an overview of the in vitro assays that have been used to study membrane active peptides. More detailed and technical descriptions of the biophysical techniques employed can be found in other excellent reviews [7,69].

### 3.1. Membrane Disruption Assays

Membrane disruption assays are used to characterise the state of membrane integrity in the presence of the membrane active peptide. These assays can be applied to any peptide, as they do not require fluorescently labelled peptides and are easy to run with basic equipment and easily available commercial reagents. Their disadvantage is that they do not directly measure cargo accumulation, but rather assay the state of the membrane.

#### 3.1.1. Bacterial Outer and Inner Membrane Permeability Assays (NPN/ONPG)

The NPN (N-phenyl-1-naphthyl amine) fluorescent probe assay is a simple and quick technique to investigate outer membrane permeabilisation in gram-negative bacteria. This assay is performed by adding NPN to a bacterial suspension, then measuring NPN uptake by the bacterial cells with a fluorescence spectrophotometer (excitation wavelength 350–360 nm, emission wavelength 420–460 nm) [37,70,71,72]. Intact outer membranes do not allow NPN uptake, but in membranes destabilised or disrupted by a permeating agent, NPN can integrate with the inner membrane phospholipid bilayer. The signal is generated by the increase in NPN fluorescence in a hydrophobic environment [37].

ONPG (o-nitrophenyl-β-D-galactopyranoside), is a substrate of β-galactosidase, a bacterial enzyme found within the cytoplasm. The substrate cannot penetrate the unperturbed bacterial inner membrane unless the membrane is disrupted. Then the substrate enters the cytoplasm and is hydrolysed by the enzyme, producing a chromogenic product. ONPG is added to a bacterial suspension of lactose permease-deficient cells (an enzyme necessary for ONPG uptake), and the hydrolysis of ONPG to ONP is measured by a spectrophotometer (absorbance at 405–420 nm) [37,49]. This assay reports on the state of the bacterial inner membrane and not the location of the cargo [37,71,72,73].

These two assays have been used to assess the membrane selective behaviour of membrane active peptides against the membranes of gram-negative bacteria; e.g., to decipher which lactoferrin-derived peptides are able to penetrate only the outer membrane or both bacterial membranes [73].

#### 3.1.2. Calcein Leakage Assay

The calcein leakage assay is used to assess the membrane disruption of synthetic lipid vesicles. These artificial vesicles mimic cell membranes and can be loaded with calcein, a fluorophore. Calcein within the vesicle is self-quenching due to its high concentration [74]. Peptide mediated membrane disruption leads to calcein released from the vesicle, decreasing its concentration and leading to the fluorescence signal [37,51,74,75,76,77,78,79,80,81] This assay is limited in its use to artificial vesicles, as these must be pre-loaded with calcein.

### 3.2. Measuring Peptides within Cells

In contrast to the assays directly measuring membrane disruption, these methods allow visualisation of the peptide itself within the cell. Whilst this is a more direct way to investigate the location of the peptide, these assays require a “probe” to be covalently conjugated to the peptide to measure it. Although there are peptides which show similar penetration when conjugated to different fluorophores [38,82], these probes (particularly specific bulky, hydrophobic fluorescent groups) may affect the behaviour of the peptide, meaning the results may not reflect the behaviour of the unconjugated peptide [22,83,84].

#### 3.2.1. Conjugation of Membrane Active Peptides with Fluorescent Molecules for Cellular Visualisation

Microscopy, using wide-field fluorescent and Confocal Laser Scanning Microscopy (CLSM) methods, has been applied in numerous studies to evaluate bacterial membrane permeation by membrane active peptides. Bacterial cells are washed and mixed with fluorescently labelled peptides [37,38,82,85,86] Excess peptide-fluorophore molecules are removed by washing and the samples are examined using CLSM or fluorescent microscopy to characterise the distribution of the membrane active peptide within the cell [37,38,82]. Moreover, the cells under examination can be counterstained with various dyes to evaluate more effectively the localisation of the peptides inside the cell. In a number of recent examples, FM4-64 (a membrane-staining dye), and trypan blue (permeated cell indicator) or SYTO9 (nucleic acid staining dye) was used to identify the periplasmic or cytosolic localisation of the CPPs in gram-negative bacteria by using fluorescent microscopy or CLSM imaging [38,82,87].

#### 3.2.2. Flow Cytometry

Flow cytometry can be used to characterise the membrane permeation efficiencies of membrane active peptides. Flow cytometry determines the permeation efficiency at the population level but cannot provide information on the localisation of the peptide within the cell [30,38,59,82,86,87,88,89].

#### 3.2.3. Real-Time Luminescence Assay

A split luciferase enzyme assay was adapted by scientists at Bicycle Therapeutics, to assess the capability of membrane active peptides to penetrate the bacterial outer membrane. This is achieved by monitoring the presence of the cargo within the periplasm as a function of enhanced luminescence. The “SLALOM” method (Split Luciferase Assay for Live monitoring of Outer Membrane transit) uses a decapeptide probe, called pep86, which can be conjugated to the membrane active peptide and *E. coli* cells transformed to express the non-functional split luciferase protein 11S. Pep86 cannot translocate across the outer membrane by itself, but conjugation to membrane active peptides enables crossing of the outer membrane. The conjugate can enter the periplasm, where it can bind to protein 11S, completing a functional luciferase enzyme. The luminescence detected is proportional to the amount of pep86 molecules entering the periplasm [36]. The advantage of this assay is that it measures compound accumulation in the periplasm, without the confounding addition of a fluorescent group.

### 3.3. Feature and Membrane Selectivity

As discussed in the introduction, there are many examples of mammalian CPPs with antimicrobial activity and conversely many AMPs which can penetrate eukaryotic cells [29,33]. When using membrane active peptides as an antimicrobial therapeutic, it is important to avoid adverse effects in mammalian membranes. The assays discussed below can be used to assess (1) the presence (or lack thereof) of antimicrobial activity and (2) the membrane selectivity or promiscuity of a membrane active peptide. The commonly used early in vitro methods for this purpose are the MIC assay, haemolysis assay, and eukaryotic cytotoxicity studies.

#### 3.3.1. Antibacterial Activity Assay

The Minimum Inhibitory Concentration assay (MIC) is used to assess the antimicrobial activity of a membrane active peptide. Bacterial cells are incubated overnight in various concentrations of the peptide of interest. The next day, growth inhibition is examined visually and the minimum concentration of the molecule which can inhibit the visible bacterial growth is assessed [37,49,73,77,78,90,91,92].

#### 3.3.2. Haemolysis Assay

The haemolysis assay is used to evaluate the cytological compatibility and initial mammalian cytotoxicity of membrane active peptides [93,94]. Peptides of interest are incubated with erythrocytes or whole blood for 30–60 min at 37 °C, and after centrifugation, the absorbance (at 400–540 nm) of supernatant is measured (the greater the haemolysis the greater the absorbance due to more haemoglobin in the supernatant), compared to 100% haemolysis obtained after incubation of blood samples with a positive control (e.g., Triton X-100) [34,37,50,78,93,95,96].

#### 3.3.3. Cytotoxicity Studies

This assay requires simple incubation of the compound with the cell line followed by colourimetric assays to assess the cell viability (typically using Tetrazolium salts (e.g., MTT, MST, WST, XTT)), which can only be enzymatically reduced by living cells [50,96,97,98,99]. Various mammalian cells can be used in cytotoxicity studies such as human somatic [97,98,99] or cancer cells [98,99], as well as animal cells [96].

### 3.4. Investigating Membrane Active Peptides with Biophysical Techniques

Gaining an understanding of how a peptide penetrates the membrane is very challenging and requires understanding the behaviour of both the peptide and the membrane during the peptide’s passage across the membrane. Biophysical methods to understand the states of these components can provide insight, when combined with other methods described above.

#### 3.4.1. Circular Dichroism (CD)

Circular Dichroism can be used to observe the secondary structure of membrane active peptides (α-helix, β-sheets, random coils), identified by their characteristic spectra under circularly polarised light. Many studies of membrane active peptides have used CD spectra to predict the secondary structure of the peptide in the presence of membrane mimics to assess how a membrane induces secondary structure changes [31,37,49,52,81,90,92,96,97]. The structural plasticity of the membrane active peptides can be tested in a range of experimental conditions (e.g., pH, temperature, vesicle composition and concentration) [100].

#### 3.4.2. Differential Scanning Calorimetry

Differential Scanning Calorimetry (DSC) is a technique which can record the phase transition and perturbations of lipid membranes with increasing temperature. It can be used to monitor how an interacting peptide affects the phase transition temperature of a membrane of specific composition. It can also give insights into how peptides deform membranes [101,102] or affect lipid packing and organisation [32]. Studies with DSC can be used to understand the molecular mechanisms that underlie cell permeation by membrane active peptides.

## 4. Variables Influencing the Biological Activity of Membrane-Active Peptides

For penetration both the peptide and the membrane should be considered. The following section explores which features of the membrane and the membrane active peptide should be considered when studying penetration.

### 4.1. Membrane Features

Membrane composition is vital for CPP or AMP membrane selectivity. In their innate immunity role, AMPs must selectively interact with the target pathogen without lysing the host’s cells. It is well established that one of the initial interactions of membrane active peptides with membranes is coulombic, between the cationic side chain of the peptide and the negatively charged phospholipid head groups of the bilayer [29,32,58,103]. It is however unclear what the next steps of membrane perturbation entail for penetration and whether these are a consequence of generalisable membrane characteristics or specific to individual membrane-peptide interactions. The interplay between the “vector” membrane active peptide and the target membrane is likely to be important in efficiently delivering the cargo across the membrane. This section discusses the chemical and physical characteristics of membranes which have been demonstrated to be important in determining whether a membrane active peptide is able to penetrate a specific bilayer.

#### 4.1.1. Phospholipid Composition and Distribution

Generally, the mammalian cell bilayer is polarised: the outer leaflet is zwitterionic consisting of mainly phosphatidyl choline (PC) while the inner leaflet contains both PC and the more negatively charged phosphatidyl serine (PS). In contrast, inner bacterial membranes contain the negatively charged phosphatidyl glycerol (PG), cardiolipin (CL) and the zwitterionic phosphatidyl ethanolamine (PE). PG and CL are found in both leaflets of the inner membrane [29]. Additionally, the outer leaflet of the gram-positive bacterial membrane contains a thick peptidoglycan layer rich in lipoteichoic acid and the outer leaflet of the outer membrane of gram-negative bacteria contains negatively charged lipopolysaccharide (LPS) [29,104].

Some membrane active peptides have been shown to interact specifically with negatively charged membrane analogues while others show interaction with zwitterionic membranes as well [31,32]. For example, penetratin, a CPP derived from a helical portion of a *Drosophila* transcription factor, interacts solely with anionic lipid vesicles whereas certain plant AMPs show interaction with anionic and zwitterionic analogues [32,52,58]. The majority of membrane active peptide research has focused on the interactions of peptides with prokaryotic (inner membrane of gram-negative and cell membrane of gram-positive bacteria) and eukaryotic cell membranes, with little investigation in how CPPs interact with and penetrate the outer membrane of gram-negative bacteria. Notable exceptions include: the use of LPS-containing micelles with synthetic membrane-active peptides to investigate how mutations to the peptide affect the thermodynamics of the peptide-membrane interaction [105] and studies in which free LPS is used to simulate how the outer membrane affects peptide folding [50,81].

#### 4.1.2. Fatty Acyl Chains

**Figure 2:**
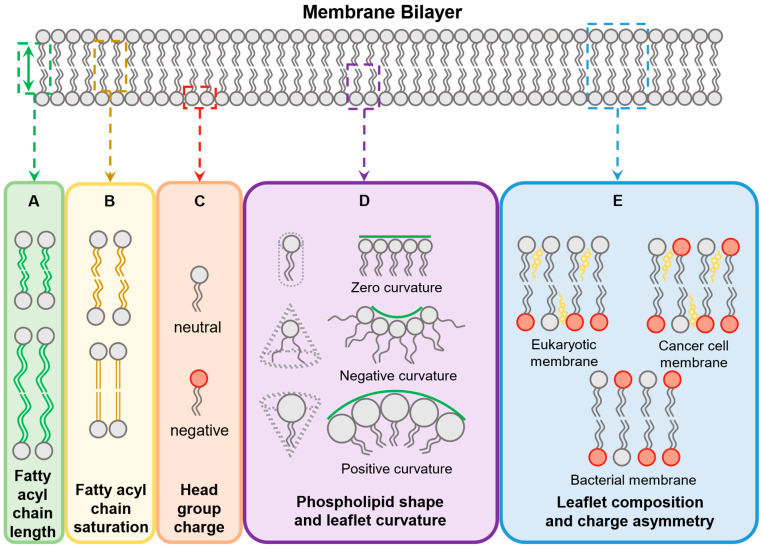
Membrane characteristics which influence penetration. Fatty acyl chain length (**A**) and saturation (**B**), as well as head group charge (**C**) affect the interaction and degree of disruption. The shape of phospholipids can also affect curvature (**D**). Leaflet composition and differences between inner and outer leaflets (**E**) also influence which membrane active peptides can penetrate a specific membrane.

The nature of the fatty acyl chains affects physical characteristics of the membrane such as fluidity, curvature (deviation from a planar configuration) and thickness (the distance between the phospholipid heads of the two membrane leaflets) [101,106]. Acyl chain length (Figure 2A) and unsaturation (Figure 2B) affect lipid thickness, fluidity [107] and packing [101,106,107]. The biophysical characteristics of a membrane are a major determinant in making them “penetrable” by membrane active peptides. In a study where binary mixtures of lipid vesicles were created, CPPs were found to interact preferentially with shorter and unsaturated phospholipids, concentrating around the CPP interaction site and causing “demixing” (phase separation of the two phospholipids). The authors postulate that this is due to looser phospholipid packing, allowing the phospholipids to laterally diffuse and aggregate to the point of contact with the CPP [32]. Short acyl chains are more prominent in prokaryotic membranes, which have a greater variation of acyl chain length and unsaturation compared to the mammalian membranes and these properties are thought to be a major reason for the selectivity observed by some AMPs. It remains unclear whether the exact acyl chain dimensions and phospholipid concentrations in a membrane would allow for penetration by a specific peptide.

#### 4.1.3. Membrane Curvature

Although initially considered a neutral feature of bilayers, membrane curvature is important in cell growth, motility, division and signaling [106]. The shape of the phospholipid (determined by the dimensions of the head and tail regions) (Figure 2D) as well as the degree of membrane protein insertion (and the shape of the inserted proteins) are major factors that affect the ability of a membrane to curve [101,106]. Induction of membrane curving is thought to be one of the major ways membrane active peptides translocate across the membrane. Internalisation of the CPP Tat was efficient in unilamellar vesicles containing PE, a phospholipid which causes local negative curvature [108]. The antimicrobial activity of NKCS has been attributed to the strong interactions of the peptide with PE-containing vesicles, where the intrinsic negative curvature of the phospholipid allows more efficient membrane disruption by the peptide [109]. The antimicrobial peptide magainin, found on the skin of the African clawed frog *Xenopus laevis*, and its derivatives MSI-78 and MSI-594, have been shown to induce positive curvatures in model membranes, leading to membrane strain, pore formation and penetration [102,110].

#### 4.1.4. Presence of Cholesterol

One of the major differences between bacterial and eukaryotic cell membranes is the presence of cholesterol, with mammalian cell membranes having between 10% and 50% [111] whilst bacterial membranes contain none (Figure 2E) [112]. It is known that cholesterol increases the mechanical rigidity of membranes [113]. This rigidity makes it more energetically expensive for AMPs to induce curvature of the membrane and it has been suggested that this forms, at least partly, the basis for AMP selectivity for bacterial rather than mammalian cell membranes [112]. In cholesterol-enriched, unilamellar vesicles, cholesterol makes penetration by synthetic and natural CPPs less efficient [79,92,114]. In contrast, in bacterial, membrane-like vesicles that contained no cholesterol, penetration was most efficient [92]. In a study where cells had their membranes treated with the cholesterol depleting agent methyl-β-cyclodextrin, cell penetration was enhanced; whereas in cells that had their cell membranes enriched with cholesterol, penetration was lowered [28].

Nevertheless, in experiments with heterogeneous, phospholipid vesicles, cholesterol does not seem to have an effect. The addition of synthetic, amphipathic magainin variants in dual-phospholipid vesicles with cholesterol has little effect on the leakage of the enclosed dye, indicating similar penetration capacities [79]. It has been suggested that heterogeneous phospholipid membranes are “raft-like” allowing phase separation between the phospholipids and formation of regions where the CPP selectively interacts with one of the phospholipids. This leads to regions of differential penetrability, nullifying the rigidity created by cholesterol [32,79,112].

#### 4.1.5. The Role of Sphingomyelin and Ceramide

Sphingomyelin is a phospholipid found in mammalian cell membranes [29]. Acid sphingomyelinase (ASMase) is an enzyme that catalyses the hydrolysis of sphingomyelin to ceramide and phosphorylcholine on the plasma membrane [115]. It has been shown that specific arginine rich CPPs are able to translocate directly through mammalian cell membranes when sphingomyelin is hydrolysed to ceramide. Inhibition of ASMase diminishes penetration while increasing the ceramide content by addition of an external source of ASMase enhances it [115,116]. It is thought that the presence of ceramide allows the creation of microdomains where CPPs can form nucleation points and directly translocate into the cell. However this process is specific to some CPPs as the sphingomyelin content of mammalian cells is variable [115,116].

#### 4.1.6. Glucosaminoglycans

Glucosaminoglycans (GAGs) are a class of anionic, polymeric carbohydrates which extend from proteins in mammalian cell membranes into the extracellular matrix surrounding cells in tissues. They have been shown to interact and influence the fluidity of membranes [117,118]. Highly anionic CPPs have been shown to interact with the GAG heparin in vitro via electrostatic interactions [11,57,119]. Unilamellar vesicles and cells which are deficient in heparin or that are treated with heparin-degrading enzymes are less penetrated by R9 [57,58,119]. It has been suggested that in high concentrations, R9 interacts with heparin increasing its local concentration near the membrane, allowing endocytosis-mediated uptake [57,58]. Therefore, when designing membrane active peptides for mammalian membranes, it is important to take into consideration both the nature of the extracellular matrix and the presence of GAGs. This method could be a way to enhance selectivity for specific cell lines or tissues.

#### 4.1.7. Contribution of Membrane Proteins

Although membrane active peptides’ mode of action is mediated by their interactions with the phospholipid bilayer, integral membrane and membrane associated proteins have an indirect role by affecting the biophysical characteristics of membranes, mainly their fluidity and curvature [106].This ability has been only infrequently studied despite proteins comprising a significant portion of all biological membranes (from 18% up to 75% by mass) [120]. Digestion of membrane proteins with trypsin in giant plasma membrane vesicles (GPMVs) has been shown to decrease the uptake of fluorescently labelled CPPs, suggesting an indirect role of membrane proteins in the penetrability of membranes [114].

The variety of different factors which affect the ability of a membrane to penetrate demands that the physicochemistry of the target membrane must be first understood to design efficacious penetrating peptides. In addition, understanding how the target membrane is differentiated from non-target membranes (e.g., targeting tumour cells only in a patient, or targeting a gram-negative strain without the membrane active peptide being cytotoxic) will allow design of peptides with higher membrane selectivity. When studying the penetrability of a specific membrane active peptide, the membrane models under which this is conducted should be as close as possible to the actual environment at which the peptide will be applied. If the application is translational, such as their use in novel antimicrobial modalities [36,37], clinical isolates of the strains targeted [36] or membrane vesicles mimicking bacterial outer membranes [81] are ideal models.

### 4.2. Peptide Features

**Figure 3:**
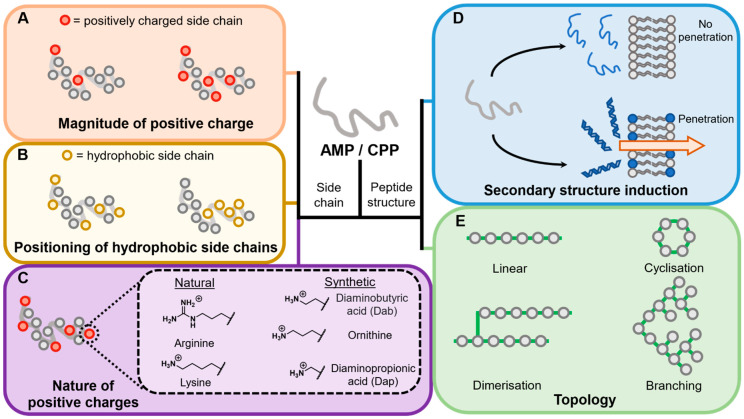
Characteristics of a peptide which affect penetrability. Positive charge (**A**), positioning of hydrophobic side chains (**B**) and the nature of positive charge (**C**) are some of the side chain characteristics that can affect penetrability. Secondary structure induction (**D**) and topology (**E**) are some of the structural characteristics that can affect penetration.

The composition of the membrane active peptide is, in part, what defines its interaction with the membrane and if that interaction leads to penetration. Multiple factors contribute to the physicochemical characteristics of a membrane active peptide (Figure 3). Below, the factors in the structure of a polypeptide which affect penetration are discussed. The effect of any of these features will be highly specific to the context of the vector peptide being used and the cargo being transported, but the aim here is to discuss features known to be important in influencing permeation properties.

#### 4.2.1. Overall Charge

Membrane active peptides are mostly cationic [7,29], (excluding a few examples [121,122]). It has been shown that the initial interaction of a peptide with membranes is electrostatic, with the positive charge of the peptide interacting with the negatively charged membrane components, such as the phospholipid heads or the LPS of bacterial outer membranes [29,32,58,103]. Studies of manually curated membrane active peptides have shown that a higher overall positive charge correlates with increased eukaryotic and prokaryotic membrane penetration [30,82,98,123] (Figure 3A). Increasing the positive charge of a peptide makes it a better penetrator as shown by peptide engineering studies [124,125] while decreasing the overall charge of a peptide with substitutions of cationic to hydrophobic amino acids decreases penetration [124,125,126]. The optimal efficiency of penetration is generally between 8 and 15 positive charges in both natural and synthetic peptides [30,124,125,126].

#### 4.2.2. Nature of Amino Acid Side Chains

##### Positively Charged Side Chains

**Figure 4:**
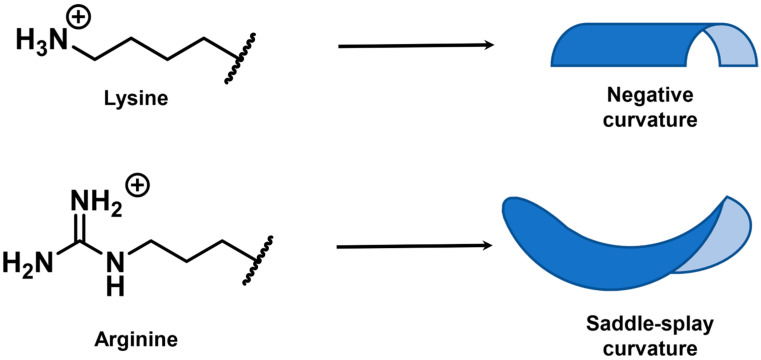
The different membrane deformations which are proposed to be caused by lysine and arginine. While lysine causes negative curvature, arginine causes a saddle splay deformation. The inner leaflet is shown in light blue while the outer leaflet is shown in dark blue.

The nature of the cationic amino acid side chain also plays a role in the penetration abilities of a membrane active peptide (Figure 3C). With regards to naturally occurring positively charged amino acids, arginine and lysine (and to an extent histidine [125,127]) have been extensively investigated and compared [38,50,125,126,128]. In specific instances [38,50,125,126,128], arginine has been shown to be more penetrative in both mammalian and prokaryotic systems compared to lysine [125,126,128]. Fluorescently tagged polyarginine CPPs show better uptake than polylysine CPPs in mammalian cells [125]. Arginine variants of the horseshow crab AMP sushi1 have shown greater penetrability and antimicrobial activity than lysine against multi-drug resistant bacterial strains [50]. Substitution of arginines with lysines in Crp4, a mouse defensin, produces lower antimicrobial activity due to negative membrane curving instead of saddle-splay curving (Figure 4) [128]. The arginine-specific saddle-splay curving has also been observed in giant membrane vesicles (GMVs) studies [22]. The authors argue that this is due to the ability of guanidium group to form multiple hydrogen bonds with phospholipids while the amino group in a lysine side chain can only form single hydrogen bonds [22]. However, arginine-to-lysine substitutions have shown no effect in the activity or membrane interactions of other defensins [128]. Alanine scanning of Tat, a fragment of an HIV transcription factor, has shown that the contribution of lysines and arginines to Tat penetrability is similar [126]. A study investigating the length and chemistry of synthetic polycationic peptides showed that dodecalysine (K12) shows marginally better penetration in *E. coli* than dodecaarginine (R12) [38]. The tri(lysine-phenylalanine- phenylalanine) (KFF)_3_ synthetic peptide has shown excellent penetrability in a variety of prokaryotic organisms [19,20,21,81,87] Therefore, these rules will be specific to the vector peptide or “cargo” being transported.

The penetrability of membrane active peptides containing non-canonical cationic amino acids has also been investigated. In some studies, synthetic Poly(ornithine) CPPs have shown more efficient penetrability for prokaryotic, but not, mammalian membranes [38,125]. However, the substitution of lysine and isoleucine to ornithine and leucine in the synthetic pepFect14(PF14) CPP indicated higher penetration efficiency in mammalian cells. When HeLa cells were transfected with PF14-splice-correcting oligonucleotide non-covalent complexes, the splice correction efficiency was enhanced compared to the lysine-isoleucine variant. This was thought to be partly due to the higher proteolytic stability of the complex [61,129]. Poly(diamonopropionic acid) (poly(Dap)) and poly(diaminobutyric Acid) (poly(Dab)) have shown enhanced penetrability compared to poly(lysine) and poly(arginine) in *E. coli*. [38] Dab is found naturally in polymyxins (cyclic non-ribosomal polypeptide-fatty acyl conjugates with general antimicrobial activity and use as last-line antibiotics) [130,131] and has been used in the design of effective murepavadin-polymyxin B_1_ conjugates for antimicrobial use [99]. The use of non-canonical amino acids also allows the investigation of the effect of side chain length on penetrability. The side chain length of lysine ((-CH_2_-)_4_) can be varied by non-canonical amino acids ornithine ((-CH_2_-)_3_), Dab((-CH_2_-)_2_) and Dap (-CH_2_-) [38,132]. In one study, increasing the length of the aliphatic side chain has shown penetration enhancement in mammalian cells [126]. For the AMP V_681_, the variation of the aliphatic side chains shows no enhancement in the antimicrobial activity against clinical isolates of the gram-negative bacterium *A. baumannii,* but Dab and Dap variants show significant enhancement in penetration selectivity (via a substantial drop in haemolytic activity). Furthermore, a study by Inoue et al. suggests that, for at least repetitive polycationic CPPs, increasing the side chain length of the amino group enhances penetration of prokaryotic membranes [38]. This effect was previously hypothesised to be due to enhanced freedom of movement of long aliphatic chains rather than the hydrophobicity of the longer side chains, although this study was conducted solely in mammalian cells [126]. In contrast, the comparison of CPPs in a study investigating the efficiency of delivering DNA into human lung fibroblasts, where lysines was substituted with ornithine, histidine or Dap, indicated that the Dap derived CPP offered a higher transfection efficiency than the ornithine or lysine derived CPPs. This was explained by the fact that the Dap containing CPPs is more sensitive to acidic pH changes which occur in the endocytotic pathway [127]. Overall, there are a multitude of natural and synthetic positively charged amino acids and varying the nature of the positive charge is an avenue worth exploring.

##### Natural Side Chain Variants

N-Methyl-variants of different amino acids show differential penetrability results. Methylation of the arginine guanidyl side chain group appears to decrease the permeation ability of octaarginine [133] while 1-methyl tryptophan decreases the anti-microbial ability of cyclic R_4_W_4_ in gram-positive bacteria as a result of lower penetrability [98].

##### Hydrophobicity

Hydrophobic amino acid side chains have also shown to be important in the ability of many membrane active peptides to cross phospholipid bilayers. The addition of phenylalanine in KFF peptides enhances the antimicrobial activity of CPP-peptide nucleic acid (PNA) conjugates, nucleic acid mimetics that are able to penetrate both bacterial membranes and induce protein expression repression by binding to mRNA molecules in a complementary manner [21]. In a study of 55 CPPs, it was shown that the most efficient CPPs contain a large amount of cationic residues and inclusion of hydrophobic residues seemed to enhance cell penetration [82]. The addition of a single tryptophan residue in nona-arginine (R9), a synthetic CPP, significantly alters its penetrability. Incubation of R9 with fluorescent, dye-containing lipidic vesicles ruptures the membrane, releasing the dye rapidly while the tryptophan-containing variant causes a gradual leakage without disrupting the shape of the vesicle [22]. The substitution of tryptophan for leucine or phenylalanine in the synthetic peptide RW9 substantially decreases its permeability [134]. The degree of hydrophobicity (number of tryptophan to phenylalanine substitutions) and the positioning of hydrophobic residues (Figure 3B) also affect penetration. The authors suggest that although arginine initiates the interaction with the membrane, tryptophan is able to insert deeper into the bilayer whereas leucine or phenylalanine residues show a more transient interaction with the membrane leading to lower penetration. The importance of tryptophan has also been displayed in structure-function study of indolicidin, a widely studied AMP. Substitution of all the tryptophans with aliphatic side chains resulted in significant reduction of the antimicrobial activity of indolicidin while tryptophan to phenylalanine substitutions showed no effect [135]. Tryptophan is also important for variants of synthetic peptide R_4_w_4_. Replacing tryptophans with any other hydrophobic residue abolishes its antimicrobial activity while increasing the number of tryptophans in cyclic variants enhances it, suggesting that hydrophobicity is important for complete translocation into cells [98]. All these studies show that tweaking hydrophobicity is a key factor to consider when trying to enhance penetrability.

#### 4.2.3. Induction of Secondary Structure

Most CPPs have a disordered/random coil conformation and no general secondary structure in solution [30,31,32,49,50,52,81,119,123]. In the presence of secondary structure inducers, such as SDS [81,136], lipidic vesicles [31,52,81] or intact bacteria [81] many CPPs adopt an alpha helical or, to a smaller extent, β-sheet structure (Figure 3D). An exception to this is the (KFF)_3_K CPP, which does not seem to change its structure [81]. Nevertheless, for most membrane active peptides, the degree of induced helicity or structural change is generally dependent on the nature of the membrane, with more anionic mixtures inducing a greater extent of conformational change than less anionic and more neutral ones (Figure 3D) [31,90]. It has been argued that the extent of a membrane active peptide’s conformational change affects its function and penetration ability. Brand et al. argued peptides that undergo a greater extent of helicity are more likely to cause large phase perturbations in a membrane and therefore more likely for the peptide to be an AMP (i.e., they kill the bacteria) [90]. Typically, more unstructured peptides tend to cause less membrane perturbations and are less likely to be an AMP [90]. In addition, a study on the effect of CPPs on lipid mixing (which has a similar phospholipid disruption profile to membrane permeation), concluded the greater the degree of structural change from a disordered conformation a peptide undergoes, the more likely it is to be a CPP [123].

#### 4.2.4. Stereochemistry

In most instances the chirality of the membrane active peptide (or individual amino acids within the sequence) does not affect function since the interaction with the membrane is non-stereospecific. Studies have shown that the *D*-variants of synthetic peptides seem to show similar if not slightly greater penetration efficiencies [16,125,126,135]. Importantly, membrane active peptides that are wholly or partially *D*-conformed also show greater resistance to proteolysis [6,21,53] which could explain their greater apparent penetrability [21,125,126,137]. Inverting or retro-inverting (inversion and inclusion of *D*-amino acids) the sequence of amino acids has also produced similar results [21,135]. Nevertheless, the use of *D*-isomers is not always conducive to penetration. Compared to the *L*-isomer, the *D*-isomer of polymyxin B nonapeptide does not allow sensitisation of bacterial outer membranes, despite the fact that its association with LPS remains unchanged between the two enantiomers [138]. It is therefore worth considering choosing an enantiomeric variant of the designed cell-penetrating peptide.

#### 4.2.5. Differential Topological Design

A substantial number of naturally occurring membrane active peptides have a linear primary sequence. It is however possible to create topologically unique peptides with additional linkages (Figure 3E). Examples of such peptides have been shown to have selectivity [48,98,139] and proteolytic resistance [98,140].

##### Cyclisation

Cyclic non-ribosomal polypeptides (e.g., polymyxins) and their derivatives are naturally occurring antimicrobial peptides which are highly potent penetrators [130]. Recently, cyclised AMP variants have shown promising results as they seem to selectively penetrate bacterial membranes without causing major damage to mammalian cells or to the host in in vivo studies [99,141]. Polymyxin derivatives which lack the fatty acyl chain have also shown to be potent bacterial outer membrane permeators [21,130,131]. Cyclic variants of CPPs have shown greater penetration compared to their linear counterparts [17,27,124,137] while also exhibiting higher stability [142]. which has been attributed to resistance to proteolytic cleavage [98,142]. A cyclised version of Tat was more efficient at delivering GFP to the cytoplasm compared to its linear counterpart [27]. Engineered cyclic variants of synthetic peptide R_4_W_4_ have shown promise as antimicrobials as they seem to selectively kill bacteria but not mammalian cells in vitro [98]. Further investigation of peptide cyclisation strategies may enable further fine-tuning of their activity and increased stability in vivo.

##### Dimerisation

sC18 is a peptide derived from the C-terminus of the antimicrobial peptide CAP18 [48,139]. Its dimerisation by the covalent addition of the N-terminus of one sC18 molecule onto the ε-amino group of a lysine at the centre of the sequence of another creates a dimer ((sC18)_2_) which has shown enhanced cell penetrating ability [139]. Interestingly, (sC18)_2_ has been shown to selectively permeate cancer cell lines more effectively than epithelial kidney cells or fibroblasts [48,139]. Despite no change in its secondary structure, this CPP is able to interact with anionic membranes seen in cancerous cells but not with zwitterionic membranes like the ones seen in normal cell lines [48]. The dimerisation of magainin and buforin, two CPPs from amphibians, show enhanced membrane vesicle disruption and membrane translocation compared to their monomeric forms [30]. These studies illustrate that dimerization might be the correct avenue in increasing penetration in many membrane active peptides.

##### Branching

Membrane active peptide dendrimers are a class of branching peptides where lysine, Dap or Dab are used as branching nodes [140]. This topology enhances proteolytic resistance: in vitro [140] and in blood serum [143], compared to its linear counterpart. The design can be customised for the specific application using combinatorial libraries [143]. Dendrimers have been created to allow for enhanced cell penetration and delivery of cargo into mammalian cells but with lower haemolytic activity [143] as well as those that have antimicrobial but not cytotoxic activity [144,145]. Antimicrobial dendrimers seem to cause higher membrane disruption than their linear counterparts [145].

When designing a membrane active peptide, all the above factors should be considered. Structure-Activity Relationship studies are important in understanding the relationship between the physicochemical characteristics of the peptide and penetration. Additionally, the penetration ability of a peptide depends on many external factors such as temperature, concentration and cell type. A study of penetratin showed that all these parameters affect the mode and degree of its penetrability [58]. The tagging of fluorophores on CPPs has been known to affect the ability of CPPs to cross a membrane [22,146]. Fluorophore conjugated R9 has been shown to cause slower leakage of lipid vesicles, indicating that the kinetics and mode of penetration are affected [22]. The covalent attachment of green fluorescent protein (GFP) onto buforin decreases its translocation efficiency [146]. It is therefore important to consider how the “payload” might also interact with the membrane active peptide and how that interaction might enhance or hamper the penetrability of the conjugate.

## 5. Use of Membrane Active Peptides as Vectors for the Development of Novel Antimicrobials

The careless overuse of antibiotics in healthcare and agriculture has led to the rise of antimicrobial resistance (AMR), making treatment of infections with current antibiotics harder. It has been estimated that, in 2019, the global death toll due to AMR was almost 5 million people [147]. The rapid advancement of AMR has led the World Health Organisation to publish a list of pathogens which pose the greatest threat to global health [148,149]. The pathogens listed have shown resistance even to the “last resort” antibiotics which are used as the last line of defence [148,150]. Therefore, there is a global urgent need for the development of new antibiotics against multi-drug resistant (MDR) pathogens. The overarching rules that govern the penetrability of membrane active peptides can act as a general guide for the design of novel antibiotics. Membrane active peptides can act vectors, delivering an antimicrobial moiety into bacterial cells. What follows are examples of research into various vector conjugates (Table 1).

One strategy employed is to covalently attach membrane active peptides to existing antibiotics to enhance their antimicrobial activity (i.e., to increase potency for a specific antibiotic or to give gram-negative activity to gram-positive-specific antibiotics) [46,151,152,153,154,155,156,157].

Vancomycin is perhaps the best-case study for the conjugation of polycationic oligopeptides. These have been shown to increase the potency of vancomycin against vancomycin-intermediate or -resistant *S. aureus.* [151,152]. In a recent study, the attachment of short poly(lysine) motifs increased the antimicrobial activity of vancomycin against many vancomycin-resistant *S. aureus* strains [151]. Furthermore, in a study where hexaarginine(R6) was conjugated to vancomycin has produced a compound that was able to show efficacy in an in vivo mouse model while showing a superior pharmacokinetic profile compared to vancomycin [152]. Conjugation of Hecate to vancomycin has also shown antimicrobial activity in gram-positive vancomycin resistant *S. aureus* without causing haemolysis [156].

On the other hand, the conjugation of polycationic moieties to existing gram-positive specific antibiotics as a way to combat gram-negative pathogens has yielded limited effectiveness [151,152,153,154]. In the two studies [151,152]. neither poly(lysine) or poly(arginine)-vancomycin conjugates showed any enhanced activity against *E. coli*. In contrast, the conjugation of a single arginine on vancomycin has shown enhanced antimicrobial activity in a panel of gram-negative pathogens [153]. However, Shi et al. illustrated that the conjugation of vancomycin to LPS-interacting membrane active peptides yielded conjugates which exhibited enhanced in vitro antimicrobial activity against pathogenic strains of, *A. baumannii*, *P. aeruginosa* and *K. pneumoniae* while also displaying low cytotoxicity against human cell lines [154].

These studies indicate the modification of vancomycin with membrane active peptides is a promising avenue for the development of new molecules. However, as with other examples, there is not a universal membrane active peptide and efficacy of this approach likely demands optimising of the vector peptide to the cargo and to the bacterial strain being targeted.

Conjugation of a variety of AMPs to antibiotics of the β-lactam and aminoglycoside class has similarly produced conjugates of varying efficacy. A study by Li et al. AMP-β-lactam conjugates showed some antimicrobial activity against *A. baumannii*, *P. aeruginosa* and *K. pneumoniae* laboratory strains but mild to no activity in clinically isolated MDR strains [157]. The conjugation of a bovine-derived AMP to a cephalosporin was considered a way to leverage β-lactams as prodrugs for the creation of antimicrobials, specifically for MDR strains which have acquired their resistance by upregulation of β-lactamases [46]. The conjugate showed moderate enhancements in antimicrobial activity for a gram-positive MRSA strain and β-lactamase positive *E. coli* strains compared to cephalosporin but is less active compared to the AMP alone [46]. In another recent study, it was shown that when magainin derivative 9P2-2 is conjugated to ampicillin, the conjugate has a higher antimicrobial activity than either of its two components alone or when added in combination. The conjugate was shown to be efficacious against laboratory *E. coli* and clinical *A. baumannii* strains but not cytotoxic to human HEK 293 cells [158]. The conjugation of penetratin derivatives to tobramycin, an aminoglycoside, has showed enhanced inner and outer membrane penetration compared to tobramycin alone. Despite this, antimicrobial activity of the conjugates was lower compared to tobramycin alone for exponentially growing cells but higher in persister cells, which have reached stationary phase and cease to replicate and grow. The authors postulate active influx mechanisms of antibiotics such as tobramycin in exponentially growing cells but not in stationary cells is one possible explanation for this discrepancy [155].

The creation of AMP-CPP conjugates has also been employed as a strategy to enhance the activity of existing AMPs. Conjugation of the nona-arginine (R9), a synthetic CPP, to the natural AMPs, magainin and M15, increased MICs against both gram-positive and gram-negative bacteria, compared to the AMPs alone. Crucially, this effect was not observed for a non-conjugated mix of the AMP and CPP. The authors attributed at least some of the benefits of conjugation to increased permeation [37]. In addition, the conjugation of Tat to KR-12 (fragment of human AMP cathelicidin) showed an enhancement of antimicrobial activity, compared to standalone K-12 or Tat, against gram-positive *S. aureus* and gram-negative *E. coli*. In addition, in vivo *S. aureus* infections of mice were treated effectively with the conjugate [159].

Conjugation of membrane active peptides to cytosolic target inhibitors, which are otherwise unable to cross the bacterial envelope has produced compounds which can act intracellularly. Patel et al. used the arginine rich Tat peptide conjugated to a series of peptidic histone deacetylase inhibitors to target *S. aureus*, *E. coli* and *P. aeruginosa*. The conjugation strategy was successful in *E. coli*, leading to ~100-fold improvement in activity compared to the unconjugated histone deacetylase inhibitor, whilst activity enhancements in *S. aureus* and *P. aeruginosa* were more modest (<10 fold). Importantly, these conjugates are not cytotoxic to mammalian cells [78]. Peptide-nucleic acids (PNAs) have also been conjugated to bacterial CPPs. PNAs are nucleic acid analogues with a peptide backbone, designed to inhibit specific bacterial gene expression and prevent bacterial growth by their mRNA or rRNA anti-sense complementation [160]. However, the large size of PNAs means entry mechanisms are required to cross the outer membrane. Conjugation of the PNA with the CPP (KFF)_3_K allowed entry of the conjugate into the cell and killing of *E. coli* cells [19,21]. Whilst a transporter (SbmA) is typically used to deliver the PNAs across the inner membrane, low MICs in a transporter knockout (ΔSbmA) strain demonstrate that vectors like (KFF)_3_K can even deliver compounds into the cytoplasm.

In the last decade, there has been a considerate amount of interest in attaching moieties to endolysins, bacteriophage hydrolases, to make them permeable to the outer membranes of gram-negative bacteria [6,161]. The AMP-endolysin SMAP29-LysPA26, derived from a sheep AMP [47], has shown moderate activity against, *E. coli, K. pneumoniae* and *P. aeruginosa* strains [162]. In addition, another conjugate, Art175, has shown moderate antimicrobial activity against multiple different *A. baumannii* strains in either exponential or stationary phases [163]. Indicating these conjugates offer a promising strategy for combatting gram-negative pathogens.

Research has also been conducted on the use of membrane active peptides as part of novel classes of antibiotics [19,20,21,36,37,87]. In recent years, several groups have attempted to use membrane active peptides as an entry mechanism to transport larger compounds into the bacterial cell. Bicycle Therapeutics, which uses phage display to generate chemically scaffolded, bi-cyclic *“Bicycle”* peptides, demonstrated that conjugation of the *Bicycle* to a AMP/CPP “vector” allowed entry of the peptide conjugate into the cell [36]. More specifically, attachment of a vector derived from the tick AMP Ixosin-B [35] to *Bicycles* allowed penetrating of the outer membrane and inhibited a periplasmic target, showing antimicrobial activity against pathogenic strains of *E. coli, P. aeruginosa* and *A. baumannii.*

However, in few of these studies was the target membrane considered. The choice of the membrane active peptide to be used should be based on the nature of the strain being targeted. Choosing AMPs which have been shown to interact with the gram-negative specific LPS [81,154] would be a first step to making sure your conjugate will have antimicrobial activity. By then conducting structure-activity studies, the specific membrane active peptide could be “tweaked” to enhance antimicrobial activity and selectivity, such as the investigation of how aliphatic primary amine side chains can affect penetration and selectivity towards a specific gram-negative bacterium [132]. The examples shown by these studies suggest that conjugation is a promising strategy for creating new classes of antibiotics and enhancing those currently available. Large and more systematic studies are required to establish which “vector” membrane active peptides should be paired with which payloads to give the optimum activity, balanced against an appropriate selectivity/cytotoxicity profile.

## 6. Conclusions

Despite extensive study, understanding of the mechanism and the factors which affect membrane permeation by membrane active peptides remains incomplete, largely due to the complexity and heterogeneity of the system. Understanding how a specific peptide interacts with the target membrane is vital for the application of this group of molecules. Importantly, the membrane to be targeted should be understood in as much detail as possible, using a variety of techniques, including the ones mentioned in this review (Section 2). Here, understanding the gram-negative outer membrane, requires understanding of how LPS interacts with membrane active peptides and how this interaction leads to membrane penetration and permeabilisation. In addition, other factors, such as membrane thickness and unsaturation (Section 3), are contributing factors in the degree of penetration. Furthermore, different gram-negative species, or even strains of the same species, contain different LPS variants [164] which should be considered as they might alter penetration efficiency. Gram-positive strains bacteria have a different membrane composition which could make a membrane active peptide-membrane interaction different, and observations made in gram-negative bacteria may not apply in gram-positive strains. As discussed in Section 4, the specific physicochemical features of the peptide should also be considered when designing novel antibiotics. Understanding a specific peptide’s mode of penetration is crucial and even the limited number of structure-activity relationship studies on membrane-active peptides reviewed here demonstrate the need for extensive optimisation of the membrane active peptide to the system. Conjugate antibiotics which contain a membrane active peptide (or fragments thereof) offer great potential as a new class of antimicrobials. An important step is to consider how the “cargo” affects the ability of the “vector” to penetrate a bacterial outer membrane and how the “vector” affects the antimicrobial activity of the “cargo”. Nevertheless, conjugates containing membrane active peptides is a promising research area for the development of novel and potent antimicrobials.

As new technologies for the discovery of antimicrobials are developed, new strategies to deliver these molecules into the cell are required. The use of membrane active peptides to deliver these molecules into cells is still underdeveloped but offers promise.

## Figures and Tables

**Table 1:** Structures and peptide sequences for the conjugates discussed in this review. “Cargo” and Vector represent the major functional moieties in the conjugates. (for clarity, linkers have been omitted).

“Cargo” *	Vector *	Reference
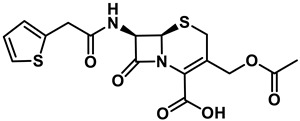 Cephalothin	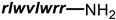 D-Bac8C(Leu^2,5^) **	[46]
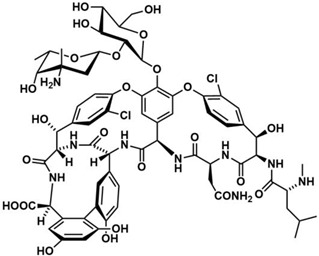 Vancomycin	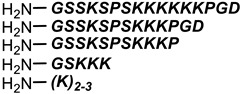 Polylysine Variants	[151]
	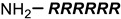 Hexa-arginine (R6)	[152]
	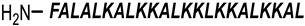 Hecate	[156]
	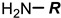 Arginine	[153]
	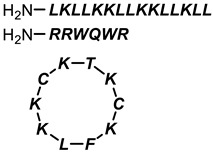 LL-15, RR-6 and cyclo-KC-10	[154]
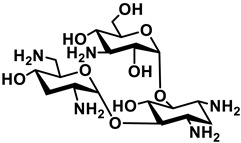 Tobramycin	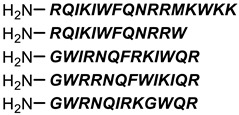 Penetratin (top) and variants	[155]
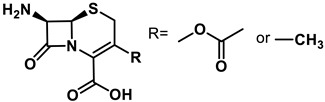 Cephalosporin core	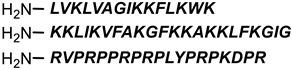 CA(1–7)M(2–9)NH2, MS1-78, Chex1-Arg20	[157]
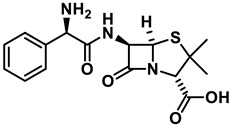 Ampicillin	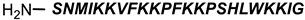 9P2-2	[158]
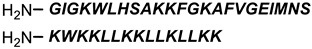 Magainin and M15	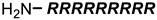 Nona-arginine (R9)	[37]
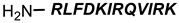 KR-12	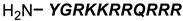 Tat peptide	[159]
Histone Deacetylase (enzyme) ***		[78]
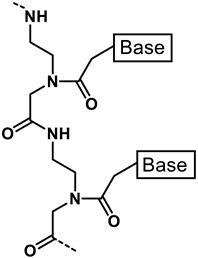 PNA	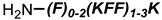 KFF motif peptide and variants	[19,21]
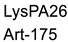 Endolysins (enzyme) ***	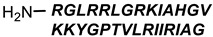 SMAP-29	[162,163]
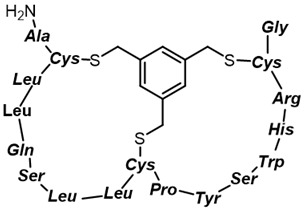 Bi-cyclic peptide (*“Bicycle”*)	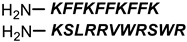 KFF motif peptide and DRAMP 1853	[36]

* Sequences highlighted in bold and are italicised represent amino acid one-letter or three-letter codes. ** The specific peptide contains D-amino acids, represented by lowercase letters. *** Names of enzymes in the conjugate.

## Data Availability

Not applicable.
